# Static Stretch Increases the Pro-Inflammatory Response of Rat Type 2 Alveolar Epithelial Cells to Dynamic Stretch

**DOI:** 10.3389/fphys.2022.838834

**Published:** 2022-04-11

**Authors:** Jorge M. C. Ferreira, Robert Huhle, Sabine Müller, Christian Schnabel, Mirko Mehner, Thea Koch, Marcelo Gama de Abreu

**Affiliations:** ^1^ Department of Anesthesiology and Intensive Care Medicine, Pulmonary Engineering Group, University Hospital Carl Gustav Carus Dresden at Technische Universität Dresden, Dresden, Germany; ^2^ Department of Anesthesiology and Intensive Care Medicine, Clinical Sensoring and Monitoring Group, University Hospital Carl Gustav Carus Dresden at Technische Universität Dresden, Dresden, Germany; ^3^ Department of Intensive Care and Resuscitation, Anesthesiology Institute, Cleveland Clinic, Cleveland, OH, United States; ^4^ Department of Outcomes Research, Anesthesiology Institute, Cleveland Clinic, Cleveland, OH, United States

**Keywords:** modulation, VILI, static stretch, dynamic stretch, alveolar epithelial cells type 2, inflammation

## Abstract

**Background**: Mechanical ventilation (MV) inflicts stress on the lungs, initiating or increasing lung inflammation, so-called ventilator-induced lung injury (VILI). Besides overdistention, cyclic opening-and-closing of alveoli (atelectrauma) is recognized as a potential mechanism of VILI. The dynamic stretch may be reduced by positive end-expiratory pressure (PEEP), which in turn increases the static stretch. We investigated whether static stretch modulates the inflammatory response of rat type 2 alveolar epithelial cells (AECs) at different levels of dynamic stretch and hypothesized that static stretch increases pro-inflammatory response of AECs at given dynamic stretch.

**Methods:** AECs, stimulated and not stimulated with lipopolysaccharide (LPS), were subjected to combinations of static (10, 20, and 30%) and dynamic stretch (15, 20, and 30%), for 1 and 4 h. Non-stretched AECs served as control. The gene expression and secreted protein levels of interleukin-6 (IL-6), monocyte chemoattractant protein-1 (MCP-1), and macrophage inflammatory protein 2 (MIP-2) were studied by real-time polymerase chain reaction (RT-qPCR) and enzyme-linked immunosorbent assay (ELISA), respectively. The effects of static and dynamic stretch were assessed by two-factorial ANOVA with planned effects post-hoc comparison according to Šidák. Statistical significance was considered for *p* < 0.05.

**Results:** In LPS-stimulated, but not in non-stimulated rat type 2 AECs, compared to non-stretched cells: 1) dynamic stretch increased the expression of amphiregulin (AREG) (*p* < 0.05), MCP-1 (*p* < 0.001), and MIP-2 (<0.05), respectively, as well as the protein secretion of IL-6 (*p* < 0.001) and MCP-1 (*p* < 0.05); 2) static stretch increased the gene expression of MCP-1 (*p* < 0.001) and MIP-2, but not AREG, and resulted in higher secretion of IL-6 (*p* < 0.001), but not MCP-1, while MIP-2 was not detectable in the medium.

**Conclusion:** In rat type 2 AECs stimulated with LPS, static stretch increased the pro-inflammatory response to dynamic stretch, suggesting a potential pro-inflammatory effect of PEEP during mechanical ventilation at the cellular level.

## Introduction

Mechanical ventilation (MV) is a lifesaving therapy in respiratory failure. During conventional MV, positive pressure is used to overcome the resistive and elastic properties of the respiratory system, leading to stretch, and strain of lung units. Stress and strain are crucial to keep the homeostasis of the lungs (Hubmayr and Kallet, 2018). Stretching of alveolar epithelial cells (AECs), also during MV, is a trigger of surfactant turnover and release (Abreu et al., 2008). However, MV may also cause injury, amplifying the lung-specific inflammatory response in both healthy and injured lungs, with release of cytokines, and damage to the alveolar integrity (Xuan et al., 2015; Rentzsch et al., 2017).

Lung injury due to MV, so-called ventilator-induced lung injury (VILI), has different pathophysiological mechanisms (Loza et al., 2015; Pelosi et al., 2018; Abreu et al., 2019). These include tidal overdistension, or volutrauma, and cyclic opening and closing of atelectatic lung units, termed atelectrauma (Rocco et al., 2012). Excessive cell stretching increases the release of surfactant proteins (Bartolák-Suki et al., 2017), production of inflammatory cytokines, and other pro-inflammatory molecules through different pathways by modulation of gene expression (Santos and Slutsky, 2000). Thereby, such events also cause numerous cellular and biochemical events in the pathogenesis of acute respiratory distress syndrome (ARDS) (Sipahi, 2014).

Mechanisms of mechano-transduction constitute the basis of the epiphenomenon generally referred to as biotrauma. In clinical practice, positive end expiratory pressure (PEEP) is used in an attempt to protect lungs from atelectrauma (Dreyfuss et al., 1988; Vlahakis et al., 1999; Huhle et al., 2018). However, PEEP strategies have yielded conflicting results regarding lung protection (Spieth et al., 2009; Bugedo et al., 2017). While the effect of PEEP on lung macrostructure is better defined, i.e., the stabilization of lung units and a more even distribution of mechanical stress (Rentzsch et al., 2017), little is known about how static stretch, resulting from use of PEEP, impacts the inflammatory response of AECs under dynamic stretching. Studies revealed that, in cultured AECs, non-variable cyclic stretch, in contrast to non-stretched resting conditions, induces the production of cytokines (Vlahakis et al., 1999; Li et al., 2003), reactive oxygen species (Chapman et al., 2005) and promotes cytoskeleton remodeling (DiPaolo et al., 2010), rendering these cells a valuable model for studying these aspects *in vitro*. Therefore, in the present study, we aimed to investigate the inflammatory response of rat type 2 AECs cell line to different conditions of stretching. We hypothesized that static stretch increases the pro-inflammatory response of AECs to dynamic stretch.

## Materials and Methods

The detailed methodology of the experiments is described in the Supplementary Figure S1.

### Cell Culture Stretch and Pre-Stretch

AECs L2 cell line CCL-149^™^ from rats (ATCC, Wesel, Germany), were grown on BioFlex six-well plates (Flexcell International Corporation, Hillsborough, United States) at a density of 1.3 × 10^5^ cells/well in DMEM (Biochrom, Berlin, Germany) containing 10% fetal bovine serum (FBS, Thermo Fisher Scientific, Bremen, Germany) and 50 μg/ml gentamycin sulfate (Biochrom, Switzerland). Cells were incubated in this medium at 37°C and 6.5% CO_2_ for a period of 24 h. After, 16–20 h before stretch experiments, cells were washed twice with sterile phosphate buffered saline (PBS) and incubated with DMEM containing 1% FBS, 50 µg gentamycin sulfate/ml, 4 mM L-alanyl*-*L*-*glutamine, and 10EU/mL FBS supplementation. Before each stretch experiment, cells were preincubated 1 h with 2 μg/ml LPS (*Escherichia coli* O111:B4, SIGMA-Aldrich, St. Louis, United States). Plates were stretched by 10, 20, or 30% static and 15, 20, and 30% dynamic stretch for 1 and 4 h. Stretching frequency was 0.5 Hz with a stretching/relaxation ratio 1:1 (sinusoidal pattern).

### Stretching Device

**FIGURE 1:**
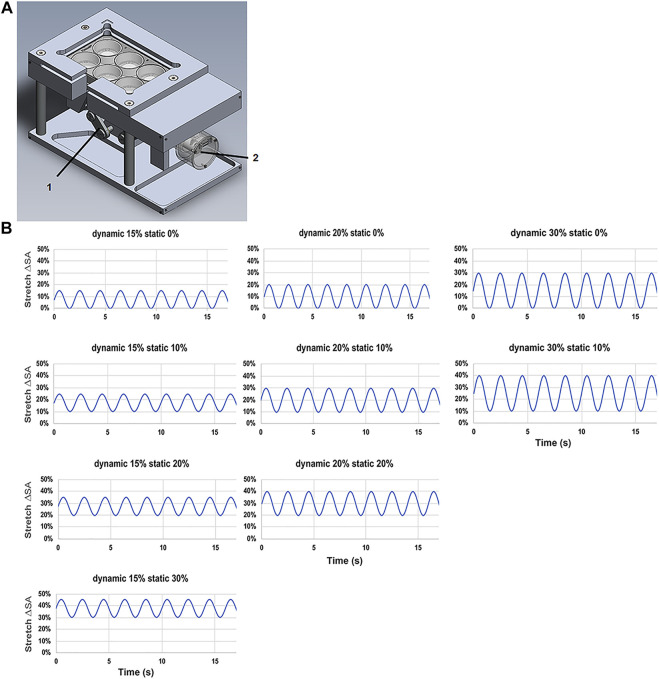
Stretching device for L2 alveolar epithelial cells. **(A)** Stretch chamber device used in the experiments with (1) brushless motor driver and (2) vertical cylindrical indenter. **(B)** Patterns of the tidal stretch ΔSA (estimated change in cell surface area) with the time (s) of L2 AECs performed on the flexible silicone elastomer membrane with a non-variable static stretch patterns of 0, 10, 20, and 30% with dynamic stretch of 15, 20, and 30%.

The custom designed and made stretching device, three cylindrical intenders were used to apply a homogeneous stretch on three membranes of a BioFlex Culture six-well plate (Flexcell International Corporation, Hillsborough, United States), while three served as non-stretched controls. A stepper motor (Maxon Motor AG, Sachsen, Switzerland) performed a vertical motion of the intender on the plate. A custom program, (LabView, National Instruments, Austix, TX, United States), was used to control the driving stepper motor and allowed the adjustment of the stretching parameters, time, frequency, the stretching amplitude (Figures 1A–B).

### Cell Viability Assay

Cell viability assay was performed using the Cytotoxicity Detection Kit Plus (Roche, Mannheim, Germany). Cell viability was assessed by measuring lactate dehydrogenase (LDH) activity in the culture supernatant.

### RNA Extraction

Total RNA was extracted using the peqGOLD Total RNA Kit (VWR, Dresden, Germany) and reverse transcribed with the qScript cDNA SuperMix (Beverly, United States). In both cases, we followed the manufacturer’s instructions.

### Gene Expression Analyses

The gene expression of amphiregulin (AREG), interleukin-6 (IL-6), chemokine (C-X-C) motif ligand 2, known as monocyte chemoattractant protein-1 (MCP-1), chemokine (C-C motif) ligand 2, known as macrophage inflammatory protein 2 (MIP-2) were detected by quantitative polymerase chain reaction (qPCR). The house-keeping genes, glyceraldehyde-3-phosphate dehydrogenase (GAPDH) and hypoxanthine-guanine phosphoribosyltransferase (HPRT) were used as controls. For Real Time PCR, PerfeCTa SYBR Green FastMix (Quanta Biosciences Inc., Gaithersburg, United States) was used. cDNA products were analyzed by semiquantitative RT-PCR using the delta delta threshold cycles CT (∆∆CT) method (Rao et al., 2013). The primers (Eurofins Genomics, Ebersberg, Germany) used are listed in Table 1. The chosen primer pairs were double-checked in the primer blast tool, to ensure specificity for the target gene.

**TABLE 1:** Primers used for quantitative polymerase chain reaction (qPCR).

Primer	Sequence (5′-3′)	Length of cDNA product	Transcriptnumber in ensembl
GAPDH s	AAC TTT GGC ATC GTG GAA GGG CT	138 bp	ENSRNOT00000050443
GAPDH as	ACC AGT GGA TGC AGG GAT GAT GTT		
HPRT s	TTT CCT TGG TCA AGC AGT ACA GCC	89 bp	ENSRNOT00000045153
HPRT as	TGG CCT GTA TCC AAC ACT TCG AGA		
IL-6 s	GAC AAA GCC AGA GTC ATT CAG AG	165 bp	ENSRNOG00000010278
IL-6 as	TTG GAT GGT CTT GGT CCT TAG CC		
MIP-2 s	AGA ACA TCC AGA GCT TGA CGG TG	108 bp	ENSRNOG00000002792
MIP-2 as	GGG CTT CAG GGT TGA GAC AAA CT		
MCP-1 s	ATG ATC CCA ATG AGT CGG CTG GAG	104 bp	ENSRNOT00000007159
MCP-1 as	GCA CAG ATC TCT CTC TTG AGC TTG		
AREG s	AAG AAT CCG TGT GCC GCC AAG TTT	124 bp	ENSRNOG0000002754
AREG as	TTT CTC CAC ACC GTT CGC CAA AGT		

qRT-PCRs were run on the PCR MyiQ™ 2 Cycler (Biorad, Kabelsketal, Germany), using the IQ 5 software (version: 2.1.97.1001). qRT-PCR was performed in triplicate cDNA samples under the following conditions: 95°C for 30 s, followed by 45 cycles at 95°C for 5 s, 58°C for 15 s, and extension at 68°C for 10 min.

### Cytokine and Chemokine Determination

Supernatant was centrifuged at 1,000 g for 5 min to remove the cells debris. Protein secretion of IL-6, MCP-1, and MIP-2 were detected using available commercial Enzyme-linked Immunosorbent Assay ELISA kits (Thermo Scientific; Invitrogen, Darmstadt, Germany).

### Immunostaining and Microscopy

First, L2 AECs were washed with phosphate buffered saline (PBS), fixed with 4% (w/v) paraformaldehyde in PBS (15 min at room temperature/RT), and washed with PBS (2 × 3 min). Afterwards, cells were permeabilized with ice-cold 0.1% (v/v) Triton X-100 in PBS (5 min, RT), washed with PBS (2 × 3 min), and then blocked with 3% (w/v) bovine serum albumin source in PBS (20–30 min, RT). Then, cells were incubated with Phalloidin-Alexa 488 (1:500) (Molecular Probes, Invitrogen Corporation, Waltham, Massachusetts, United States), which labels filamentous actin, or with a primary antibody against Zonula Occludens Protein (ZO-2) (1:300) (#Sc-8148 Santa Cruz Biotechnology Inc., Dallas, United States), or anti-Surfactant Protein C (1:200) (#AB3786 Millipore Corporation, United States) a specific L2AECs marker, 1 h at RT, washed with PBS (2 × 3 min), and labelled with the secondary antibody Alexa-Fluor-488 goat anti-rabbit (1:500) (Thermo Fischer Scientific, Waltham, Massachusetts, United States) and DAPI (1:200) (Sigma-Aldrich Chemie GmbH, Munich, Germany), during 30 min at room temperature. Subsequently, cells were washed with PBS (2 times × 3 min) and mounted on microscope slides using MOWIOL (Calbiochem/Merck, Darmstadt, Germany). L2 AECs were imaged using an Olympus SD-OSR Spinning Disc Confocal Microscope equipped with a 60x numerical aperture N.A. = 1.4 oil DIC objective (Carl Zeiss Microimaging, Jena, Germany) at the Imaging Facility “Medizinisch -Theoretisches Zentrum” at Technical Dresden University, Z-stacks were acquired with 1 µm or 30 µm intervals between consecutive focal planes. Images were analyzed, processed, and quantified with ImageJ (Rasband and ImageJ, 2021), and Acrobat Photoshop^®^ 2022 (Adobe, United States). Actin filament orientation was analyzed using the OrientationJ Plugin (Rezakhaniha et al., 2012) and their intensity and length, as well as tight junction intensity, were assessed using FiloQuant (Jacquemet et al., 2017; Jacquemet et al., 2019). The 3D renditions were obtained from 1 µm Z-sections using the ImageJ 3D Viewer plugin (volume view) (Rasband and W.S.; Schneider et al., 2012; Schindelin et al., 2015).

### Statistical Analysis

Data is given as mean ± standard deviation (SD), unless otherwise indicated. Comparisons among groups were conducted with three-way general linear model ANOVA for factors condition (LPS, Stretch, LPS + Stretch), static (0, 10, 20, and 30%) and dynamic stretch (15, 20, 30%). Main effects for each model were assessed as marginal means (Dessau and Pipper, 2008; Lenth, 2016) with *p*-value adjustment according to Šidák for planned comparisons: for each dynamic stretch between available static stretches; and for each combination of static and dynamic stretch levels between LPS + non-Stretch and LPS + Stretch. Cell survival was tested using logistic binomial GLM with factors group (Control, Stretch, LPS or LPS + Stretch), time, dynamic and static stretch. All statistics were performed using R Statistical Programming Language (R Core Team, 2021). Statistical significance was accepted at *p* < 0.05.

## Results

### Cell Typification

The lineage of type 2 AECs was confirmed by immunostaining with an antibody against Surfactant Protein C (SP-C), a specific marker for these cells (Supplementary Figure S2).

### *In vitro* Stretch, Tight Junctions, and Cell Viability

**FIGURE 2:**
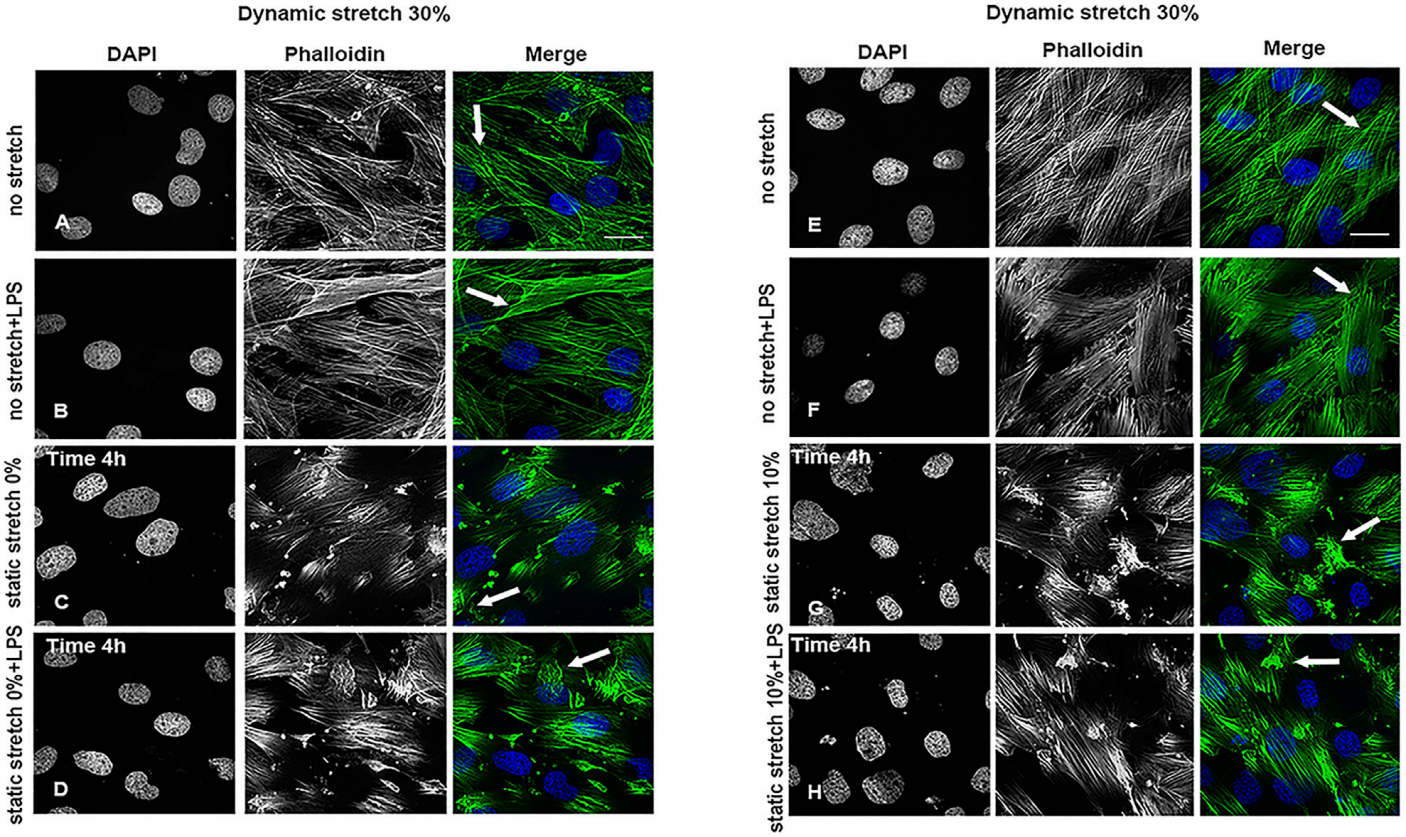
Cytoskeleton organization in L2 AECs undergoing stretch, with and without LPS for 4 h. Arrows show changes in actin filament organization in cells undergoing a 30% dynamic stretch and **(A,E)** non-stretch, **(B,F)** non-stretch + LPS, **(C)** 0% static stretch, **(D)** 0% static stretch + LPS, **(G)** 10% static stretch, or **(H)** 10% static stretch + LPS. Cells were fixed and analysed by confocal microscopy. Single channels are displayed in grey scale for DAPI (DNA) and phalloidin (actin filaments); Merge: phalloidin (green) and DAPI (blue). Data are displayed as projections of 1 μm Z-sections (*n* = 3). Scale bars: 30 μm.

As shown in Supplementary Figures S3–S5, and Supplementary Figure S8 the static and dynamic stretch did not affect the organization or intensity of ZO-2-positive intercellular tight junctions in any of the studied experimental conditions. Cytoplasmic actin filaments were intact at a dynamic stretch of 15% (Supplementary Figure S6) or 20% (Supplementary Figure S7). In contrast, a significant reduction in actin filament length, an increase in their intensity, and a change in their orientation, i.e., more actin bundles (Figure 2C, D, G, H), compared to the parallel filaments observed in control cells (Figures 2A, B, E, F), were seen at a dynamic stretch of 30% (Supplementary Figures S9–S11).

The cell survival of L2 AECs submitted to 1 h or 4 h of static and dynamic stretch was not affected by any of the respective conditions (Supplementary Figures S12A–C).

### Gene Expression of Cell Mechanical Stress and Pro-Inflammatory Cytokines

**FIGURE 3:**
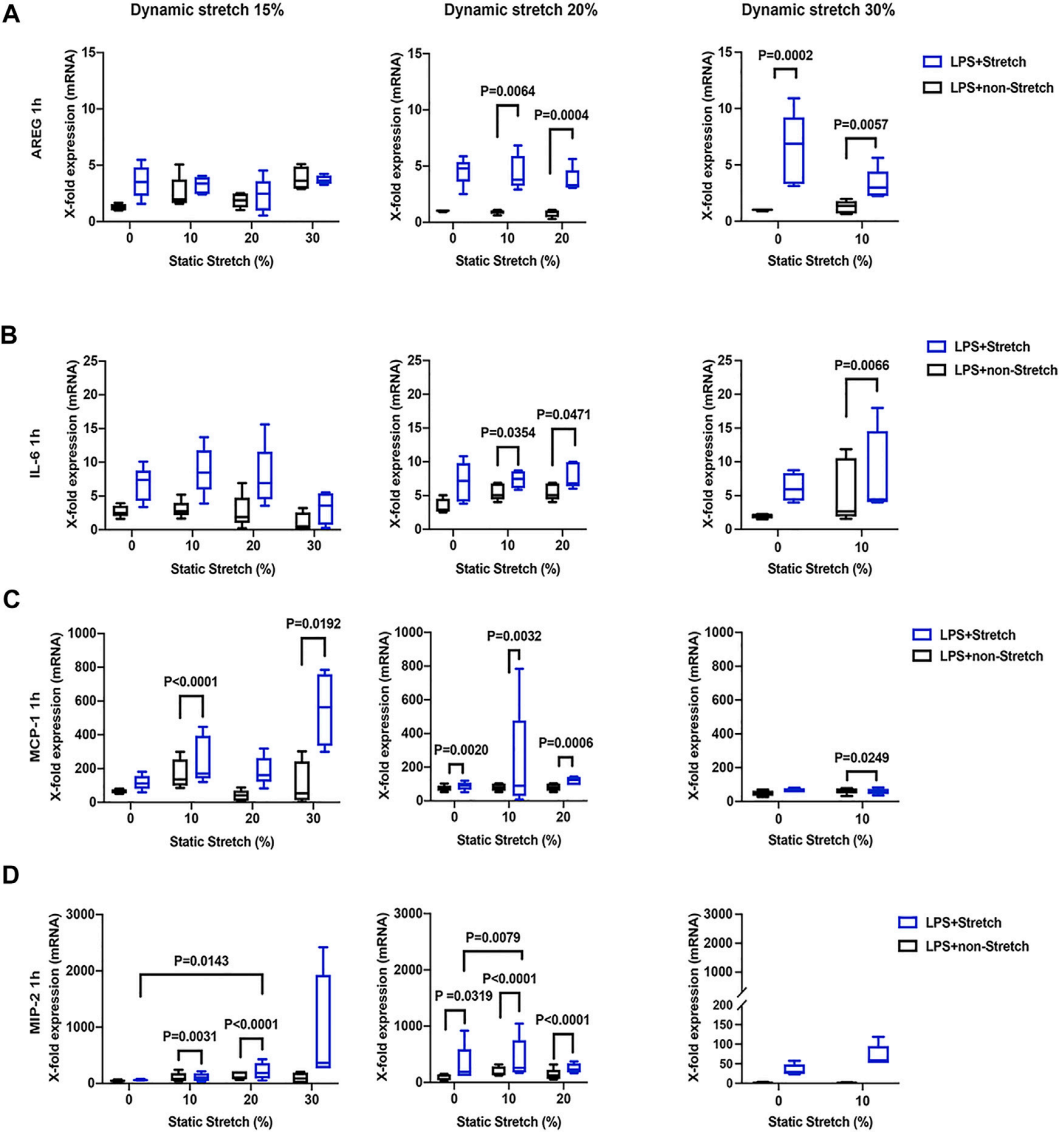
Gene expression of AREG, IL-6, MCP-1, and MIP-2 in L2 alveolar epithelial cells treated with LPS and undergoing stretch for 1 h. **(A)** Amphiregulin (AREG), **(B)** Interleukin-6 (IL-6), **(C)** monocyte chemoattractant protein-1 (MCP-1), and **(D)** macrophage inflammatory protein 2 (MIP-2). mRNA was isolated from cells treated as indicated and analyzed by real-time polymerase chain reaction (RT-qPCR). Data represents the X-fold expression of mRNA compared to control non-treated cells (*n* = 4). Results were normalized to control non-stretch, non-stimulated cells. Differences were considered statistically significant at *p* < 0.05.

**FIGURE 4:**
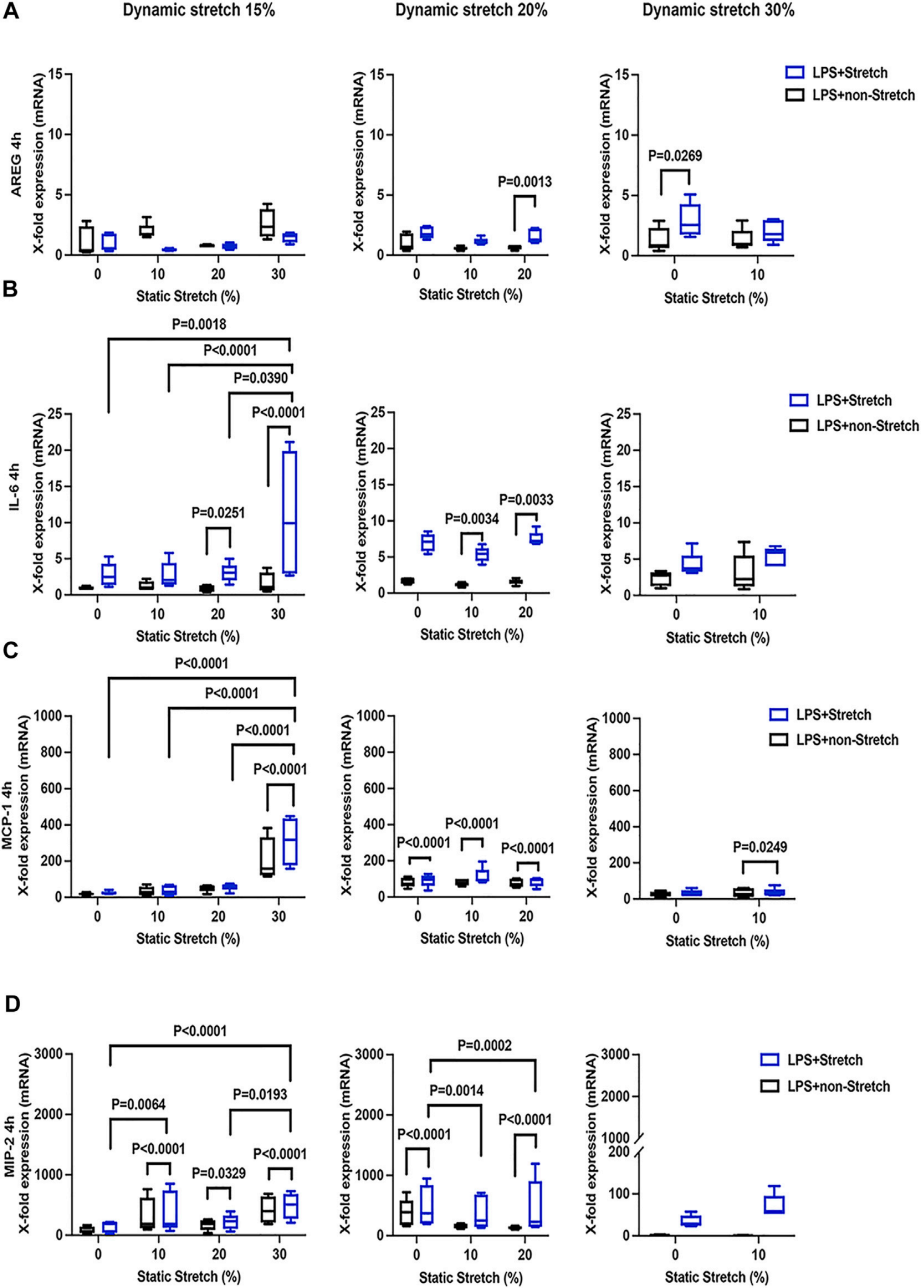
Gene expression of AREG, IL-6, MCP-1, and MIP-2 in L2 alveolar epithelial cells treated with LPS and undergoing stretch for 4 h. **(A)** Amphiregulin (AREG), **(B)** Interleukin-6 (IL-6), **(C)** monocyte chemoattractant protein-1 (MCP-1), and **(D)** macrophage inflammatory protein 2 (MIP-2) mRNA was isolated from cells treated as indicated and analyzed by real-time polymerase chain reaction (RT-qPCR). Data represents the X-fold expression of mRNA compared to control non-treated cells (*n* = 4). Results were normalized to control non-stretch, non-stimulated cells. Differences were considered statistically significant at *p* < 0.05.

The expression of AREG was significantly increased in LPS-stimulated cells under stretch (*p* < 0.05), when compared with non-stimulated L2 AECs (Figure 3A, Figure 4A). AREG expression was lower at 4 h than at 1 h in LPS-treated cells, in all studied conditions. Dynamic, but not static, stretch increased AREG expression, irrespective of the duration of experiments or LPS stimulation. The expressions of IL-6 and MCP-1, but not MIP-2, were higher in LPS stimulated cells under different combinations of static and dynamic stretch, compared to non-stimulated, cells (Figures 3B–D; Figures 4B–D). Stretch duration did not affect the expression of IL-6 or MCP-1, but the expression of MIP-2 was higher at 4 h (*p* < 0.05) compared with 1 h. All data points were normalized to non-stretched, non-LPS treated cells.

### Protein Concentrations of Pro-Inflammatory Cytokines in the Medium

**FIGURE 5:**
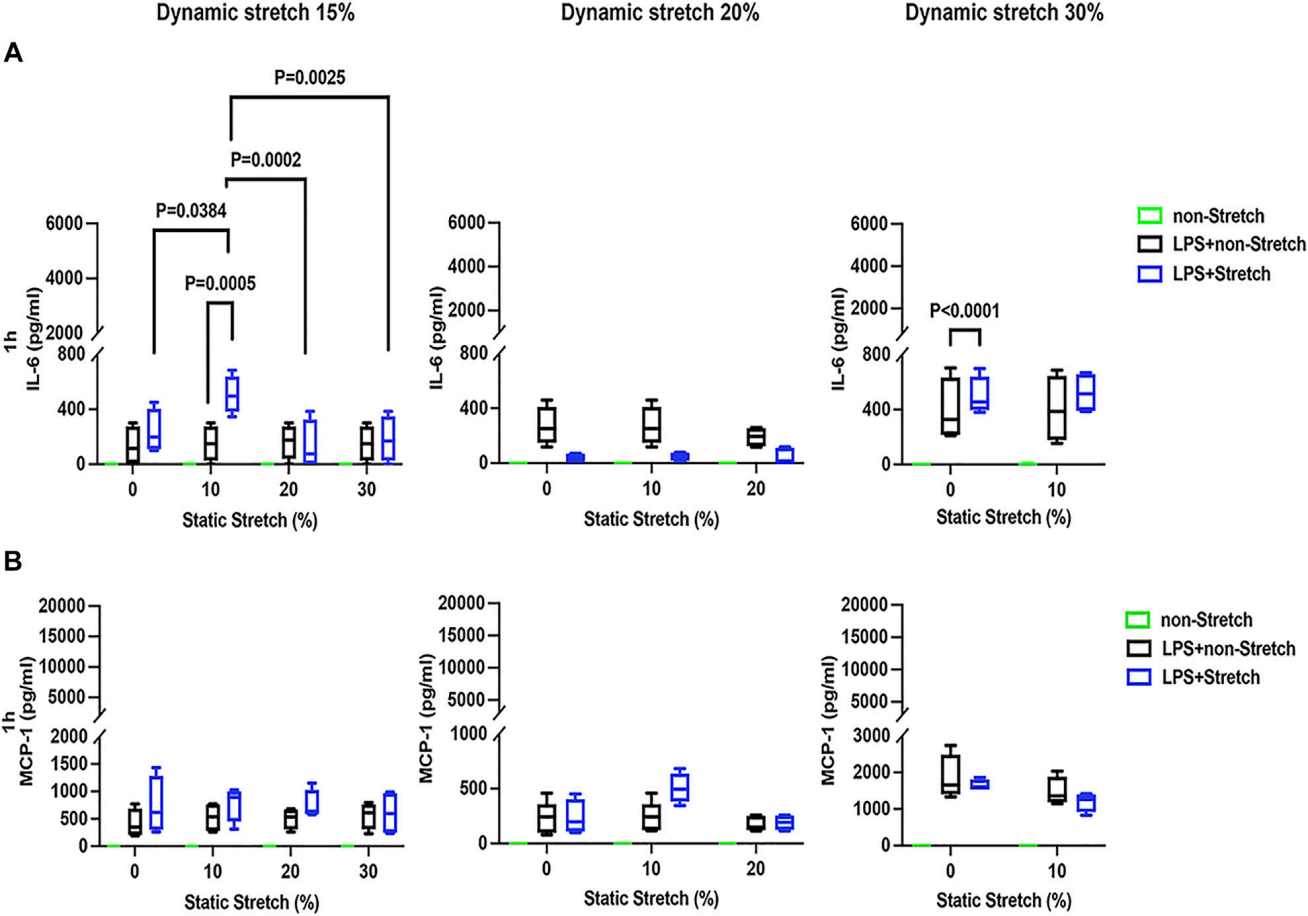
IL-6 and MCP-1 secretion in L2 alveolar epithelial cells treated with LPS and undergoing stretch for 1 h. Cells were treated, stretched with different static, and dynamic conditions 1 h, and then culture supernatants were collected: **(A)** Interleukin-6 (IL-6) and **(B)** monocyte chemoattractant protein-1 (MCP-1) protein levels were evaluated using Enzyme-linked Immunosorbent Assay (*n* = 4). Differences were considered statistically significant at *p* < 0.05.

**FIGURE 6:**
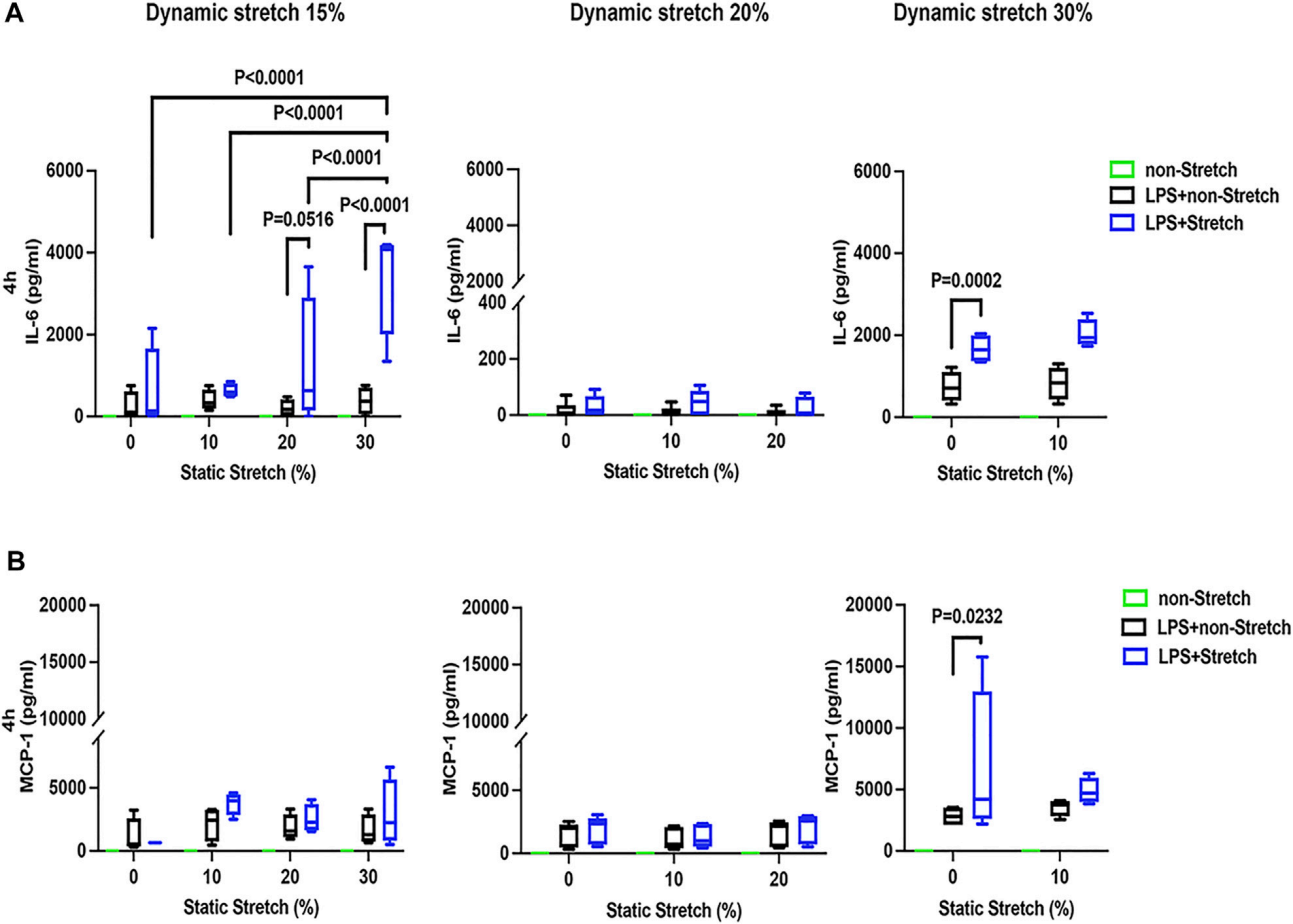
IL-6 and MCP-1 secretion in L2 alveolar epithelial cells treated with LPS and undergoing stretch for 4 h. Cells were treated, stretched with different static and dynamic conditions 4 h, then culture supernatants were collected: **(A)** Interleukin-6 (IL-6) and **(B)** monocyte chemoattractant protein-1 (MCP-1) protein levels were evaluated using Enzyme-linked Immunosorbent Assay (*n* = 4). Differences were considered statistically significant at *p* < 0.05.

In LPS-stimulated cells, the release of the pro-inflammatory cytokines IL-6 and MCP-1 was generally higher under different combinations of static and dynamic stretch, compared to non-stretch L2 AECs (*p* < 0.05) (Figures 5, 6). The secreted protein concentrations of IL-6 and MCP-1 were higher at 4 h than at 1 h (*p* < 0.001). In contrast to IL-6 release, which was increased by both static and dynamic stretch (*p* < 0.05), MCP-1 secretion was only increased by dynamic (*p* < 0.05), but not by static stretch. MIP-2 protein levels were below the detection level in the analysed medium (Supplementary Figure S13).

## Discussion

The main findings of this study were that, in LPS-stimulated, compared to non-stimulated rat type 2 AECs: 1) dynamic stretch increased the expression of AREG, IL-6, MCP-1, MIP-2, as well as the protein concentration of IL-6 and MCP-1 in the medium; 2) static stretch increased gene expression of MCP-1 and MIP-2, but not AREG, and resulted in higher protein secretion of IL-6, but not MCP-1. To our knowledge, this is the first study addressing the effects of static stretch on the pro-inflammatory response of *in vitro* L2 AECs submitted to dynamic stretch.

Our experiments combining static stretch with different dynamic stretch conditions were conducted in type L2 AECs, because they can easily grow under controlled conditions, and are the main cell type involved in the immune response of the lung alveolar epithelium (Kang and Kim, 2017). A major strength of our study is that neither dynamic nor static stretch, nor the LPS used for stimulation, generated any significant changes in cell survival, thus allowing us to directly assess the pro-inflammatory response. Importantly, static and dynamic stretch were designed to mimic the mechanical stress patterns that AECs are submitted to *in vivo*, during MV, since there is a considerable body of evidence that mechano-transduction plays a key role in VILI (Chen et al., 2018)**.** Changes in alveolar epithelial cell deformation during modifications of lung volume have been described *in vivo* (Bartolák-Suki et al., 2017). Studies in isolated lungs suggest that, if the lung volume increases by 40–100% of the total lung capacity, the basal surface area of alveolar epithelial cells increases by 34–35% (Tschumperlin and Margulies, 1999; Tschumperlin et al., 2000; Wirtz and Dobbs, 2000).

On the other hand, if a single alveolus or even the whole lung is modelled as a sphere, it can be shown that an increase of alveolar surface area (SA) by factor *a*, yields an increase of corresponding volume *V* by *a* ^3/2^. Therefore, during spontaneous breathing in humans, at a functional residual capacity (FRC) of 30 ml/kg body weight, a tidal volume of 7 ml/kg body weight results in an increase of lung volume of 23% and an increase of ∼15% of ASA. To increase lung volume from FRC to lung total capacity (TLC) (80 ml/kg body weight) would correspond to a 166% volume increase and a 92% increase of SA. In rats, however during anesthesia a FRC of 11 ml/kg has been reported (Schulz and Muhle, 2000). Ventilation with a tidal volume of 6 ml/kg body weight results in a 54% increase of LV and a 30% increase of SA. Accordingly, an increase of lung volume from FRC to TLC (42 ml/kg body weight in rats) yields a volume increase of 270% and an increase of SA of 140%. Thus, in our experiments a maximal total increase of SA of 45% mirrored dynamic and static mechanical strain occurring during low to low tidal volume ventilation in rats at low PEEP.

Mechanical ventilation may induce an inflammatory response in the lung tissue (Santos and Slutsky, 2000), leading to ARDS (Birukova et al., 2012; Sipahi, 2014). In ARDS patients, high levels of PEEP are used to reduce VILI (Briel et al., 2010). Mechanical ventilation with low tidal volumes and higher PEEP reduced the incidence of pulmonary complications in patients without acute lung injury (Neto et al., 2012). In contrast, other clinical studies have indicated that the level of PEEP is unrelated to the mortality of the ARDS patients (Walkey et al., 2017; Group et al., 2020). Recent data have also shown that higher PEEP promoted higher lung inflammation than lower PEEP, at comparable low tidal volumes and driving pressures (Ding et al., 2013; Ochiai, 2015; Hamlington et al., 2016; Silva et al., 2016). For this reason, we have asked if static stretch (which *in vivo* is increased by PEEP) modulates the inflammatory response. To mimic in AECs the pathophysiology of clinical ARDS (Costa et al., 2017), we used LPS, frequently utilized to induce inflammation *in vitro* (Dreyfuss et al., 1988; McRitchie et al., 2000; Birukova et al., 2012; Wong and Johnson, 2013) or ARDS*,* in rats, *in vivo* (Pugin et al., 1998; Ding et al., 2013).

We found that, neither dynamic stretch, static stretch, nor LPS significantly affected cell junctions and cell monolayer organization, as previously shown (Rentzsch et al., 2017; Knudsen and Ochs, 2018). Actin filament organization was not modified at lower dynamic stretch (Rentzsch et al., 2017; Knudsen and Ochs, 2018) but only at a 30% dynamic stretch, which corresponds to a high increase in total lung capacity. Previous studies have shown that MV modulates the lung inflammatory response, either through direct cell rupture or through the so-called “mechano-transduction” mechanism, which is not yet fully understood (Santos and Slutsky, 2000; Sutherasan et al., 2014; Tian et al., 2016). Recent data (Tian et al., 2016) suggest that long term (28–72 h) stretching or LPS exposure of cultured human pulmonary artery endothelial cells affect cell junctions and disrupted cell monolayers, a process mediated by RhoA kinase (Marshall et al., 2010). This is in agreement with the changes in actin filament organization we observed at a high dynamic stretch, changes that could be mediated by Rho GTPase (i.e., RhoA) signaling, and could involve crosstalk with extracellular matrix–dependent integrin signaling via SRC (Tehrani et al., 2007). Future studies are necessary to clarify these aspects.

AREG, a protein with tissue protective effects during inflammation (Zaiss et al., 2013), and is involved in the response to mechanical loading in various mesodermal-derived tissues (Deacon and Knox, 2010; Marshall et al., 2010; Shao and Sheng, 2010). We found that AREG expression was increased in LPS-stimulated cells submitted to different dynamic stretch conditions, at 1 h. However, further stimulation (4 h) reduced AREG expression, suggesting that its tissue protective effects may be reduced after long-term stretching. This decrease in AREG expression may be due to reduced transcription, and/or increased mRNA degradation (Nakayama et al., 2013), controlled by cell signaling pathways, as previously observed in other systems (Zhang et al., 2009; Terry et al., 2010; Keren, 2011; Latasa et al., 2012; Zaiss et al., 2013).

The focus of our study was to understand if and how PEEP modulated the inflammatory response (Rentzsch et al., 2017; Knudsen and Ochs, 2018). We have found that, in LPS-stimulated L2 AECs, dynamic and static stretch levels influenced both the expression and secretion of specific cytokines (i.e. IL-6, MCP-1, and MIP-2). This is in agreement with *in vivo* studies in mice, showing increased expression and release of IL-6 (Tremblay et al., 2002; Heise et al., 2011; Goldman et al., 2014), MCP-1 or MIP-2 (Hegeman et al., 2010; Hawwa et al., 2011; Herbert et al., 2016; Jia et al., 2016; Zhu et al., 2016) during MV. Specifically, in LPS-stimulated L2 AECs, the expression of IL-6, MCP-1, and MIP-2 was significantly increased after static stretch, compared to non-LPS treated cells. Our data are in agreement with *in vivo* studies showing that the expression of several cytokines (including IL-6, MCP-1, or MIP-2) was significantly enhanced after exposure to LPS (Heise et al., 2011) and that, mechanical ventilation synergistically amplified the release of IL-6, MIP-2, IL-1β, and TNF-α in rats treated with LPS (Pugin et al., 1998; Ding et al., 2013). In addition, our data shows that MCP-1 behaves differently than the other cytokines, i.e., its release from LPS-treated cells was enhanced by dynamic, but not by static stretch, suggesting that the two types of stretch may differentially influence the immune response. Moreover, these findings support previous evidence that plasma membrane tension affects the release of inflammatory cytokines, by orchestrating complex aspects of cell trafficking and motility (Keren, 2011), and modulating cell signaling (Tremblay et al., 1997; Ranieri et al., 1999).

### Possible Implications of the Findings

Our findings indicate that the mechanical tension produced by static stretch of AECs, which for example results from the use of PEEP, affects the cellular signalling pathways of inflammation. Therefore, physicians should be aware that not only macro structural changes of lungs that result from PEEP, but also microstructural changes that modulate cellular stretching might play a role in VILI. This finding supports the use of lower PEEP levels in patients with mild lung injury and ventilated with a low protective tidal volume, a concept known as “permissive atelectasis” (Guldner et al., 2016; Pelosi et al., 2018).

### Limitations

Our study holds several limitations. Firstly, *in vitro* conditions do not fully reproduce the complex environment of the lung parenchyma. However, none of the available ARDS animal models replicates the complex pathophysiological changes seen in patients, although, similarly to our model, they were essential for advancing the knowledge in the field (Spieth et al., 2009; Rentzsch et al., 2017; Braune et al., 2019). Secondly, to mimic inflammation, we have used LPS, a complex compound that may contain bacterial DNA, lipo-proteins, *etc.*, and thus has a high cytotoxicity. However, LPS is a valuable inflammatory inducer, largely used in this type of studies. Thirdly, the induced stretch transmitted through an elastic membrane to the epithelial cells differs significantly from *in-vivo* situations, where the stretching force is applied through epithelial cells onto the extra-cellular matrix.

## Conclusion

Static stretch increased the pro-inflammatory response of dynamically stretched LPS-stimulated type L2 AECs which is suggestive of a potential pro-inflammatory effect of PEEP during mechanical ventilation at the cellular level.

## Data Availability

The datasets presented in this study can be found in online repositories. The names of the repository/repositories and accession number(s) can be found in the article/Supplementary Material.
