# Supplementary material for: Static Stretch Increases the Pro-Inflammatory Response of Rat Type 2 Alveolar Epithelial Cells to Dynamic Stretch
Source: Front Physiol. 2022 Apr 11;13:838834. doi: 10.3389/fphys.2022.838834 (PMC9035495; doi:10.3389/fphys.2022.838834)
Supplement: Supplementary file 1 [file Image12.pdf]

Supplementary Material

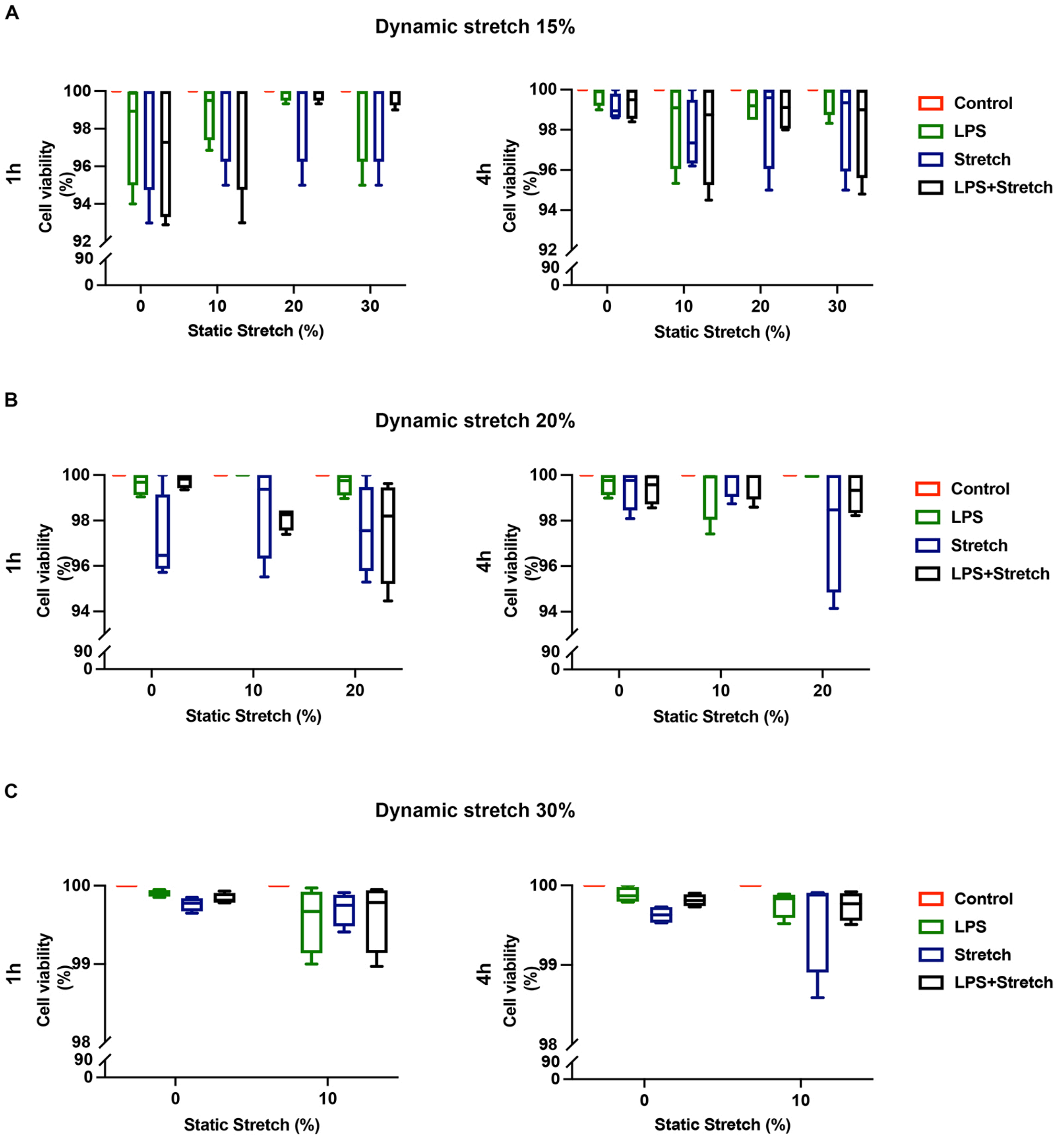

Supplementary Figure 12. Cell viability in alveolar epithelial cells type 2. Cells were exposed to different dynamic stretch (15%, 20% and 30%) and static (10%, 20% and 30%) stretch for 1h and 4h

with and without LPS stimulation (2µg/ml), as described. Cell survival was not different between the different experimental groups.
