# Supplementary material for: Static Stretch Increases the Pro-Inflammatory Response of Rat Type 2 Alveolar Epithelial Cells to Dynamic Stretch
Source: Front Physiol. 2022 Apr 11;13:838834. doi: 10.3389/fphys.2022.838834 (PMC9035495; doi:10.3389/fphys.2022.838834)
Supplement: Supplementary file 3 [file Image9.pdf]

Supplementary Material

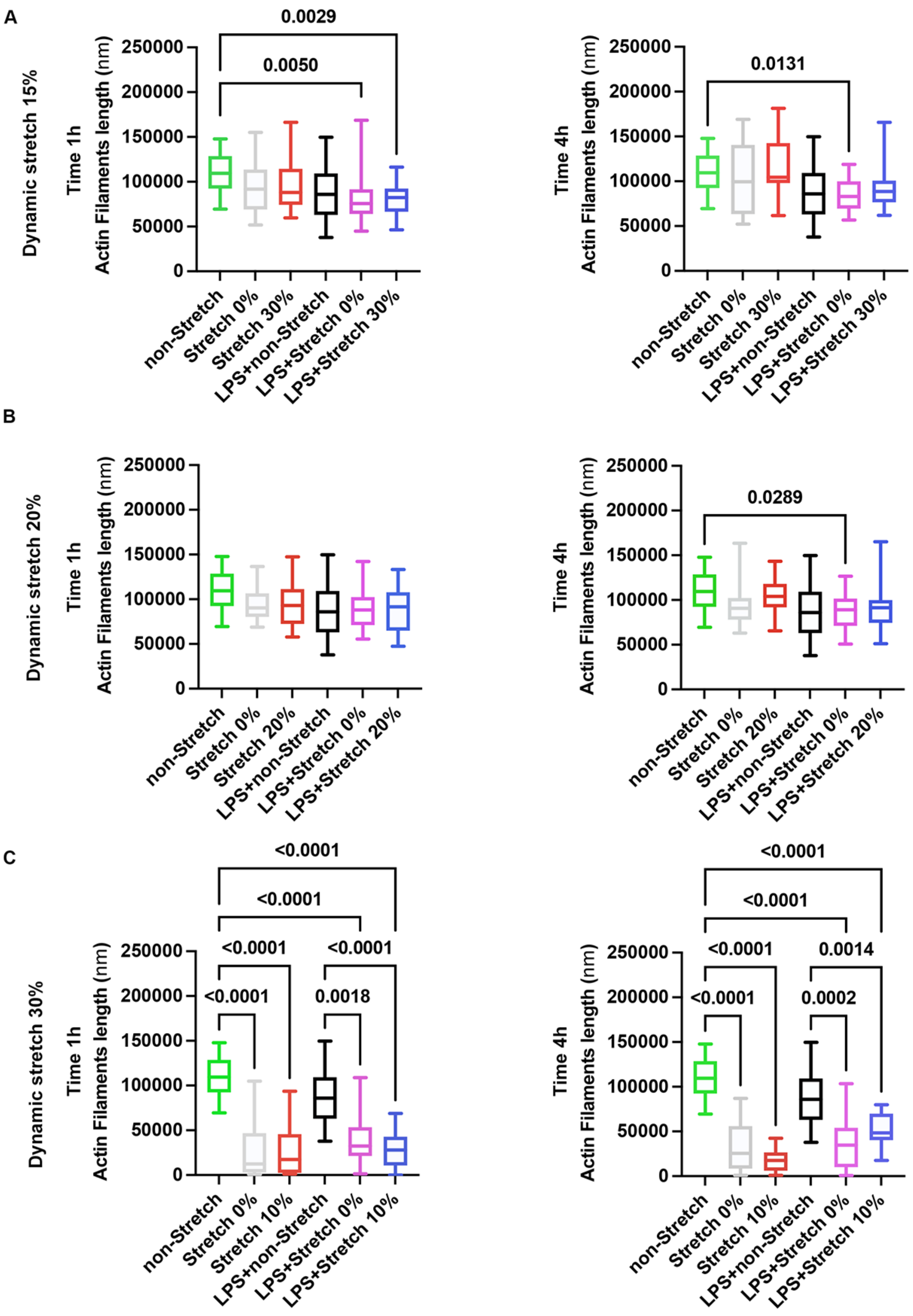

Supplementary Figure 9. Quantification of the actin filament length in alveolar epithelial cells type 2 exposed to different dynamic and static stretch conditions. (A) represents dynamic stretch 15%, (B) dynamic stetch 20% and (C) dynamic stretch 30%. Cells were fixed, stained and imaged by confocal fluorescence microscopy. Images were quantified using ImageJ and the FiloQuant plugin. Significance was observed in cells under dynamic stretch of 30% with a static stretch 0% and 10% at 1h and 4h in the conditions stretch, and stretched with LPS treatment when compared with the correspondent controls . Data are presented as mean±SD (n=3).
