# Supplementary material for: Static Stretch Increases the Pro-Inflammatory Response of Rat Type 2 Alveolar Epithelial Cells to Dynamic Stretch
Source: Front Physiol. 2022 Apr 11;13:838834. doi: 10.3389/fphys.2022.838834 (PMC9035495; doi:10.3389/fphys.2022.838834)
Supplement: Supplementary file 5 [file Image6.pdf]

Supplementary Material

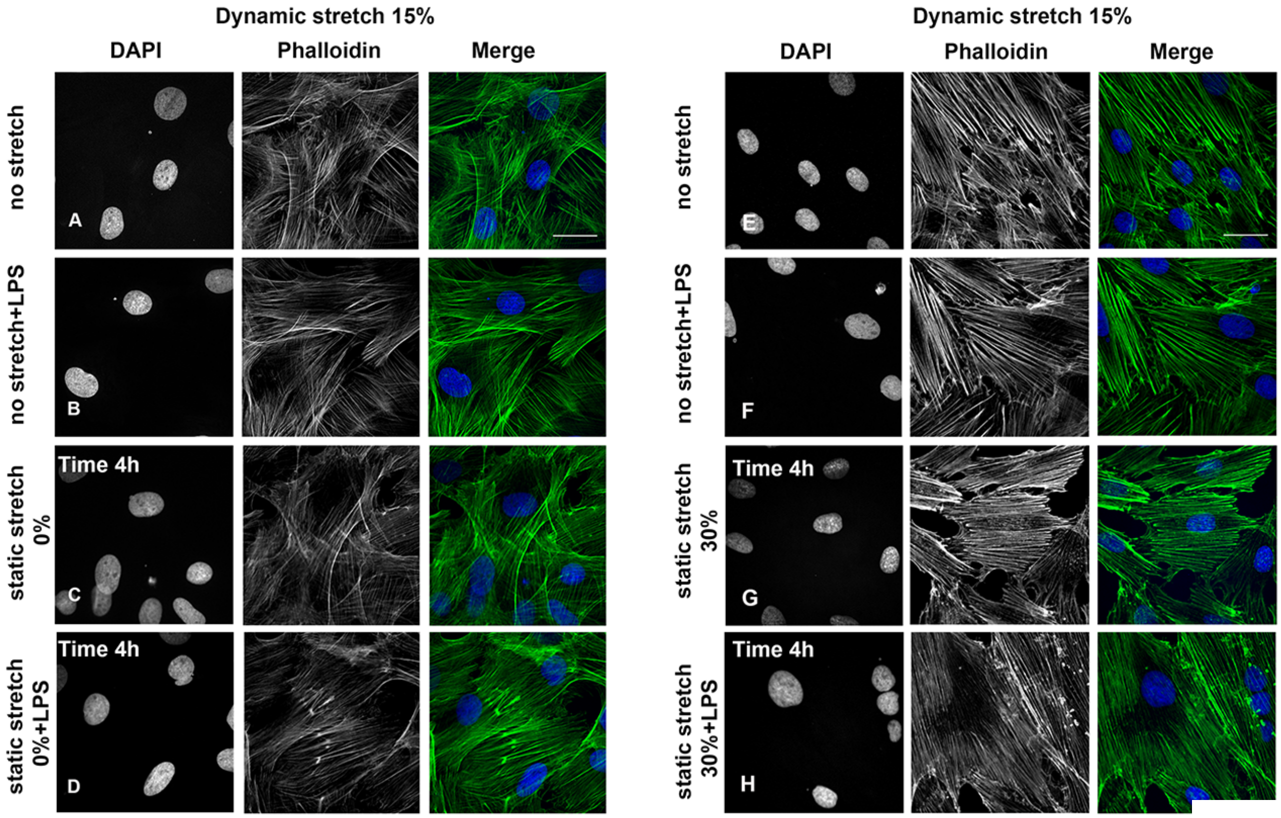

Supplementary Figure 6. Effect of static stretch combined with a dynamic stretch of 15% on L2 AECs with and without LPS. Cells were treated, fixed, stretched during 4h, stained with phalloidin (actin filaments) and DAPI (DNA), then analysed by confocal fluorescence microscopy. Data are displayed as a projection of Z-sections. Images of the first and second columns are single channels in grey scale for DAPI and phalloidin; third column: Merge: DAPI (blue) and phalloidin (green) respectively. Data are displayed as a projection of 1µm Z-sections. Scale bars: 30µm.
