# Supplementary material for: Static Stretch Increases the Pro-Inflammatory Response of Rat Type 2 Alveolar Epithelial Cells to Dynamic Stretch
Source: Front Physiol. 2022 Apr 11;13:838834. doi: 10.3389/fphys.2022.838834 (PMC9035495; doi:10.3389/fphys.2022.838834)
Supplement: Supplementary file 6 [file Image13.pdf]

Supplementary Material

Dynamic stretch 15%

% stretch	Sample/ Well	MIP-2 (mg/ml)
0-15% 1h	Control/ 2	0,058
	LPS /3	0,752
	Stretch/ 4	0,904
	LPS Stretch/ 5	1,27
	Control/ 2	0
	LPS /3	0,177
	Stretch/ 4	0
	LPS Stretch/ 5	0,238
	Control/ 2	0
	LPS /3	0
	Stretch/ 4	0
	LPS Stretch/ 5	0
	Control/ 2	0,39
	LPS /3	0,387
	Stretch/ 4	0,382
	LPS Stretch/ 5	0,332
	Control/ 2	0,341
	LPS /3	0,317
	Stretch/ 4	0,328
	LPS Stretch/ 5	0,359
0-15% 4h	Control/ 2	0
	LPS /3	3,296
	Stretch/ 4	0
	LPS Stretch/ 5	0,058
	Control/ 2	0,57
	LPS /3	4,841
	Stretch/ 4	0,207
	LPS Stretch/ 5	13,381
	Control/ 2	0,315
	LPS /3	0,286
	Stretch/ 4	0,28
	LPS Stretch/ 5	0,308
	Control/ 2	0,394
	LPS /3	0,359
	Stretch/ 4	0,4
	LPS Stretch/ 5	0,424
	Control/ 2	0
	LPS /3	1,094339623
	Stretch/ 4	0
	LPS Stretch/ 5	3,448467967
10-25 1h	Control/ 2	0,117
	LPS /3	0,238
	Stretch/ 4	0,238
	LPS Stretch/ 5	0,57
	Control/ 2	0,147
	LPS /3	0,207
	Stretch/ 4	0
	LPS Stretch/ 5	0,058
	Control/ 2	0
	LPS /3	0
	Stretch/ 4	0
	LPS Stretch/ 5	0
	Control/ 2	0
	LPS /3	0
	Stretch/ 4	0
	LPS Stretch/ 5	0
	Control/ 2	0,426
	LPS /3	0,433
	Stretch/ 4	0,448
	LPS Stretch/ 5	0,449
10-25 4h	Control/ 2	0
	LPS /3	0,298
	Stretch/ 4	0
	LPS Stretch/ 5	2,496
	Control/ 2	0
	LPS /3	19,188
	Stretch/ 4	0
	LPS Stretch/ 5	24,892
	Control/ 2	0
	LPS /3	0,298
	Stretch/ 4	0
	LPS Stretch/ 5	2,496
	Control/ 2	0,389
	LPS /3	0,386
	Stretch/ 4	0,399
	LPS Stretch/ 5	0,393
20-35 1h	Control/ 2	0,388
	LPS /3	0,388
	Stretch/ 4	0,058
	LPS Stretch/ 5	0,692
	Control/ 2	0
	LPS /3	0
	Stretch/ 4	0
	LPS Stretch/ 5	0,51
	Control/ 2	0,385
	LPS /3	0,344
	Stretch/ 4	0,378
	LPS Stretch/ 5	0,411
	Control/ 2	0,337
	LPS /3	0,433
	Stretch/ 4	0,379
	LPS Stretch/ 5	0,395
	Control/ 2	0,431
	LPS /3	0,431
	Stretch/ 4	0,406
	LPS Stretch/ 5	0,469
20-35 4h	Control/ 2	0
	LPS /3	1,668
	Stretch/ 4	0
	LPS Stretch/ 5	3,203
	Control/ 2	0
	LPS /3	0,6
	Stretch/ 4	0,058
	LPS Stretch/ 5	3,573
	Control/ 2	0,334
	LPS /3	0,396
	Stretch/ 4	0,346
	LPS Stretch/ 5	0,349
	Control/ 2	0,433
	LPS /3	0,429
	Stretch/ 4	0,433
	LPS Stretch/ 5	0,413
	Control/ 2	0
	LPS /3	0
	Stretch/ 4	0
	LPS Stretch/ 5	0
30-45 1h	Control/ 2	0,147
	LPS /3	0,358
	Stretch/ 4	0
	LPS Stretch/ 5	0,328
	Control/ 2	0,117
	LPS /3	0
	Stretch/ 4	0
	LPS Stretch/ 5	0,147
	Control/ 2	0
	LPS /3	0
	Stretch/ 4	0
	LPS Stretch/ 5	0
	Control/ 2	0,279
	LPS /3	0,295
	Stretch/ 4	0,268
	LPS Stretch/ 5	0,306
	Control/ 2	0,346
	LPS /3	0,317
	Stretch/ 4	0,384
	LPS Stretch/ 5	0,372
30-45 4h	Control/ 2	1,24
	LPS /3	1,423
	Stretch/ 4	0,238
	LPS Stretch/ 5	2,526
	Control/ 2	0
	LPS /3	0,087
	Stretch/ 4	0
	LPS Stretch/ 5	1,392
	Control/ 2	0,337
	LPS /3	0,323
	Stretch/ 4	0,34
	LPS Stretch/ 5	0,354
	Control/ 2	0,238
	LPS /3	0,141
	Stretch/ 4	0,247
	LPS Stretch/ 5	0,25
	Control/ 2	0
	LPS /3	0
	Stretch/ 4	0
	LPS Stretch/ 5	0

Dynamic stretch 20%

% stretch/time	Sample/ Well	MIP-2 (mg/ml)
0-20 1h	Control/ 2	0,262
	LPS /3	0,266
	Stretch/ 4	0,228
	LPS Stretch/ 5	0,285
	Control/ 2	0,365
	LPS /3	0,352
	Stretch/ 4	0,42
	LPS Stretch/ 5	0,369
	Control/ 2	0,383
	LPS /3	0,368
	Stretch/ 4	0,371
	LPS Stretch/ 5	0,342
	Control/ 2	0,372
	LPS /3	0,332
	Stretch/ 4	0,385
	LPS Stretch/ 5	0,347
	Control/ 2	0
	LPS /3	0
	Stretch/ 4	0
	LPS Stretch/ 5	0
0-20 4h	Control/ 2	0,117
	LPS /3	0
	Stretch/ 4	0,298
	LPS Stretch/ 5	0
	Control/ 2	4,748
	LPS /3	4,996
	Stretch/ 4	0
	LPS Stretch/ 5	10,486
	Control/ 2	0,308
	LPS /3	0,407
	Stretch/ 4	0,372
	LPS Stretch/ 5	0,296
	Control/ 2	0,586
	LPS /3	0,314
	Stretch/ 4	0,568
	LPS Stretch/ 5	0,35
	Control/ 2	0
	LPS /3	0
	Stretch/ 4	0
	LPS Stretch/ 5	0
10-30 1h	Control/ 2	3,185
	LPS /3	0
	Stretch/ 4	0
	LPS Stretch/ 5	1,297
	Control/ 2	0
	LPS /3	0
	Stretch/ 4	3,573
	LPS Stretch/ 5	0
	Control/ 2	0
	LPS /3	0
	Stretch/ 4	0
	LPS Stretch/ 5	0
	Control/ 2	7,383
	LPS /3	0,47
	Stretch/ 4	2,231
	LPS Stretch/ 5	0
	Control/ 2	0
	LPS /3	0
	Stretch/ 4	0
	LPS Stretch/ 5	0
10-30 4h	Control/ 2	3,744
	LPS /3	19,906
	Stretch/ 4	0,695
	LPS Stretch/ 5	19,542
	Control/ 2	0
	LPS /3	6,798
	Stretch/ 4	0
	LPS Stretch/ 5	9,705
	Control/ 2	0
	LPS /3	1,273
	Stretch/ 4	1,62
	LPS Stretch/ 5	7,614
	Control/ 2	0
	LPS /3	0,955
	Stretch/ 4	0
	LPS Stretch/ 5	5,031
	Control/ 2	0,329
	LPS /3	0,312
	Stretch/ 4	0,302
	LPS Stretch/ 5	0,321
20-40 1h	Control/ 2	0
	LPS /3	0
	Stretch/ 4	0
	LPS Stretch/ 5	0
	Control/ 2	0
	LPS /3	0
	Stretch/ 4	1,484
	LPS Stretch/ 5	2,645
	Control/ 2	0
	LPS /3	0
	Stretch/ 4	0
	LPS Stretch/ 5	1,733
	Control/ 2	0
	LPS /3	0
	Stretch/ 4	1,437
	LPS Stretch/ 5	0,252
	Control/ 2	0
	LPS /3	0
	Stretch/ 4	0
	LPS Stretch/ 5	0
20-40 4h	Control/ 2	0
	LPS /3	40,851
	Stretch/ 4	0
	LPS Stretch/ 5	45,073
	Control/ 2	0
	LPS /3	17,567
	Stretch/ 4	1,055
	LPS Stretch/ 5	22,315
	Control/ 2	0
	LPS /3	2,625
	Stretch/ 4	0
	LPS Stretch/ 5	7,614
	Control/ 2	0
	LPS /3	4,176
	Stretch/ 4	5,227
	LPS Stretch/ 5	4,008
	Control/ 2	0
	LPS /3	0
	Stretch/ 4	0
	LPS Stretch/ 5	0

Dynamic stretch 30%

% stretch	Sample/ Well	MIP-2 (mg/ml)
0-30 1h	Control/ 2	0,443
	LPS /3	0,388
	Stretch/ 4	0,428
	LPS Stretch/ 5	0,425
	Control/ 2	0,384
	LPS /3	0,403
	Stretch/ 4	0,42
	LPS Stretch/ 5	0,408
	Control/ 2	0,414
	LPS /3	0,366
	Stretch/ 4	0
	LPS Stretch/ 5	12,519
	Control/ 2	0
	LPS /3	8,554
	Stretch/ 4	1,824
	LPS Stretch/ 5	9,846
	Control/ 2	0
	LPS /3	16,462
	Stretch/ 4	4,112
	LPS Stretch/ 5	11,429
0-30 4h	Control/ 2	0,392
	LPS /3	0,398
	Stretch/ 4	0,433
	LPS Stretch/ 5	0,432
	Control/ 2	0,438
	LPS /3	0,416
	Stretch/ 4	0,394
	LPS Stretch/ 5	0,437
	Control/ 2	0,405
	LPS /3	0,422
	Stretch/ 4	3,284
	LPS Stretch/ 5	5,466
	Control/ 2	0
	LPS /3	0,782
	Stretch/ 4	0
	LPS Stretch/ 5	9,737
	Control/ 2	6,304
	LPS /3	8,473
	Stretch/ 4	1,195
	LPS Stretch/ 5	8,716
10-40 1h	Control/ 2	3,001
	LPS /3	7,648
	Stretch/ 4	0
	LPS Stretch/ 5	9,445
	Control/ 2	2,325
	LPS /3	9,122
	Stretch/ 4	4,293
	LPS Stretch/ 5	9,686
	Control/ 2	0
	LPS /3	4,053
	Stretch/ 4	7,483
	LPS Stretch/ 5	11,586
	Control/ 2	0
	LPS /3	7,943
	Stretch/ 4	7,065
	LPS Stretch/ 5	15,94
	Control/ 2	0,255
	LPS /3	0,271
	Stretch/ 4	0,233
	LPS Stretch/ 5	0,254
10-40 4h	Control/ 2	7,786
	LPS /3	11,898
	Stretch/ 4	10,8
	LPS Stretch/ 5	10,958
	Control/ 2	5,617
	LPS /3	6,39
	Stretch/ 4	9,043
	LPS Stretch/ 5	17,504
	Control/ 2	0,32
	LPS /3	1,32
	Stretch/ 4	0,332
	LPS Stretch/ 5	1,327
	Control/ 2	0,19
	LPS /3	0,465
	Stretch/ 4	0,439
	LPS Stretch/ 5	0,471
	Control/ 2	0,45
	LPS /3	0,428
	Stretch/ 4	0
	LPS Stretch/ 5	2,649

minimum detectable
dose = 0,5-2,7pg/ml

Rat MIP-2 ELISA Kit novex life technologies
REF: KRC1021 LOT 1780097C

Supplementary Figure 13. Effect of dynamic and static stretch on the secretion of MIP-2 at the time 1h and 4h in alveolar epithelial cells type 2. Cell supernatants were analysed for macrophage inflammatory protein 2 (MIP-2) by Enzyme-linked Immunosorbent Assay (ELISA) Kits (n=4 per group). The table shows that the protein secretion levels in the medium of MIP-2 were below the detection level in all stretch conditions.
