# Supplementary material for: Static Stretch Increases the Pro-Inflammatory Response of Rat Type 2 Alveolar Epithelial Cells to Dynamic Stretch
Source: Front Physiol. 2022 Apr 11;13:838834. doi: 10.3389/fphys.2022.838834 (PMC9035495; doi:10.3389/fphys.2022.838834)
Supplement: Supplementary file 7 [file Image8.pdf]

Supplementary Material

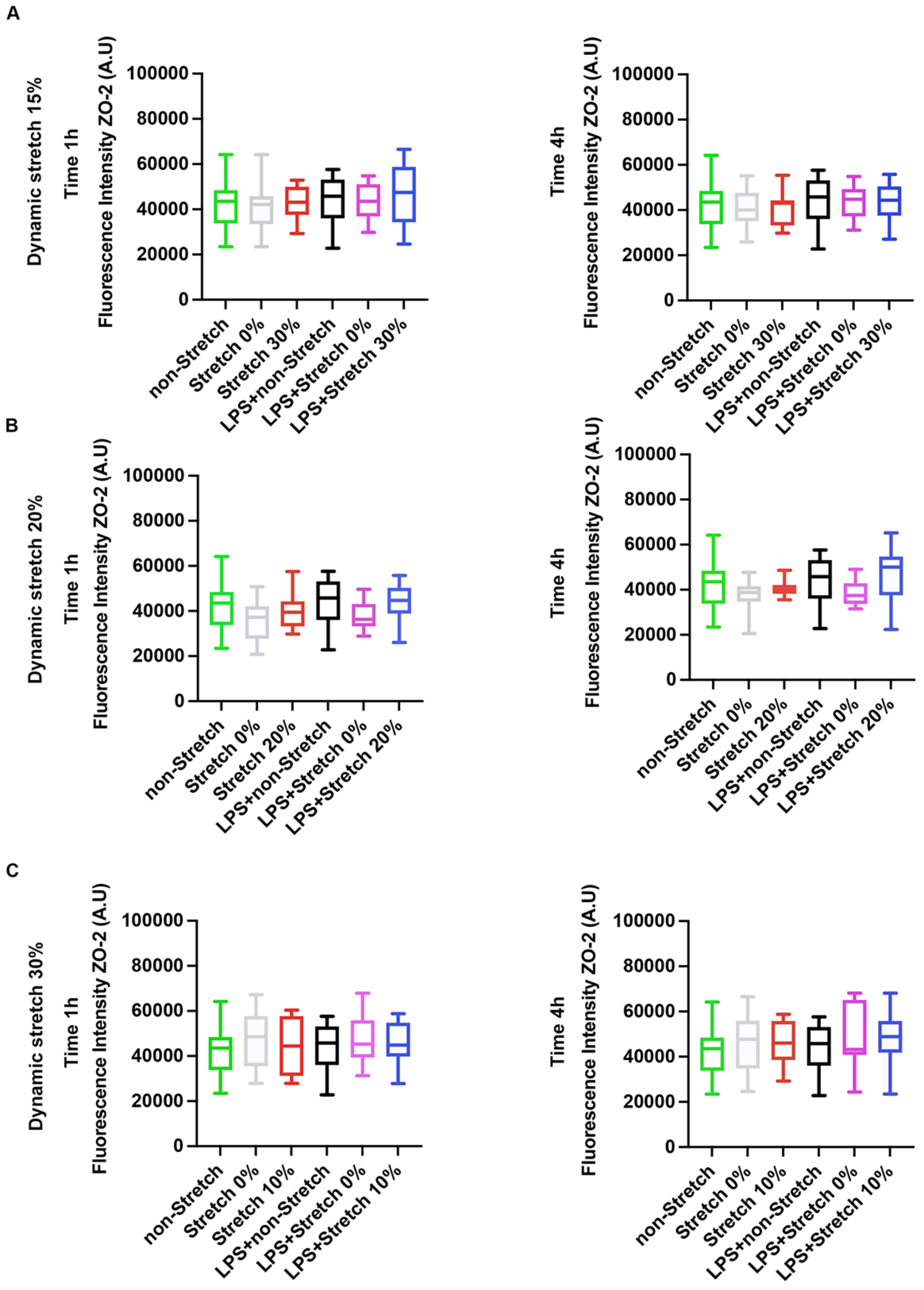

Supplementary Figure 8. Fluorescence intensity of tight junctions, Zonula Occludens Protein (ZO-2), in alveolar epithelial cells type 2 exposed to different dynamic and static stretch conditions. (A) represents dynamic stretch 15%, (B) dynamic stetch 20% and (C) dynamic stretch 30%. Cells were fixed, stained, and imaged by confocal fluorescence microscopy. Images were quantified using ImageJ and the FiloQuant plugin. Significance was not observed in any stretch condition at 1h or 4h. Data are presented as mean±SD (n=3).
