# Supplementary material for: Static Stretch Increases the Pro-Inflammatory Response of Rat Type 2 Alveolar Epithelial Cells to Dynamic Stretch
Source: Front Physiol. 2022 Apr 11;13:838834. doi: 10.3389/fphys.2022.838834 (PMC9035495; doi:10.3389/fphys.2022.838834)
Supplement: Supplementary file 8 [file Image4.pdf]

Supplementary Material

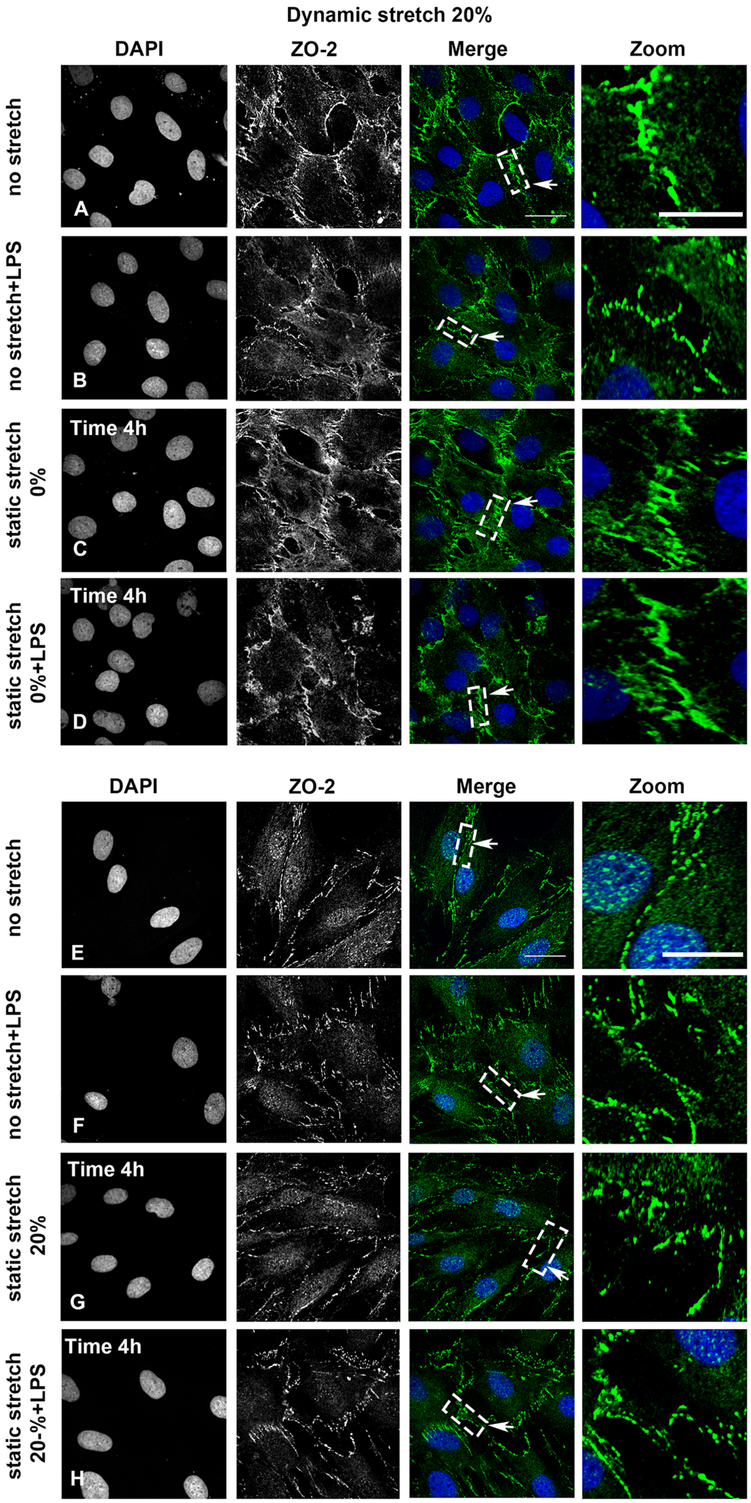

Supplementary Figure 4. Effect of static stretch combined with a dynamic stretch of 20% at 4h on L2 AECs with and without LPS. Cells were treated, fixed, stretched during 4h, stained with an antibody against zonula occludens (ZO-2) and DAPI (DNA), then analysed by confocal fluorescence microscopy. Data are displayed as a projection of Z-sections. Images of the first and second columns are single channels in grey scale for DAPI and ZO-2; third and fourth column: Merge: DAPI (blue) and ZO-2 (green) respectively. The “Zoom” panel shows a higher magnification of the region delimitated by the white box. Scale bars 30μm (zoom-out view) and 1μm (magnification).
