# Supplementary material for: Static Stretch Increases the Pro-Inflammatory Response of Rat Type 2 Alveolar Epithelial Cells to Dynamic Stretch
Source: Front Physiol. 2022 Apr 11;13:838834. doi: 10.3389/fphys.2022.838834 (PMC9035495; doi:10.3389/fphys.2022.838834)
Supplement: Supplementary file 9 [file Image2.pdf]

Supplementary Material

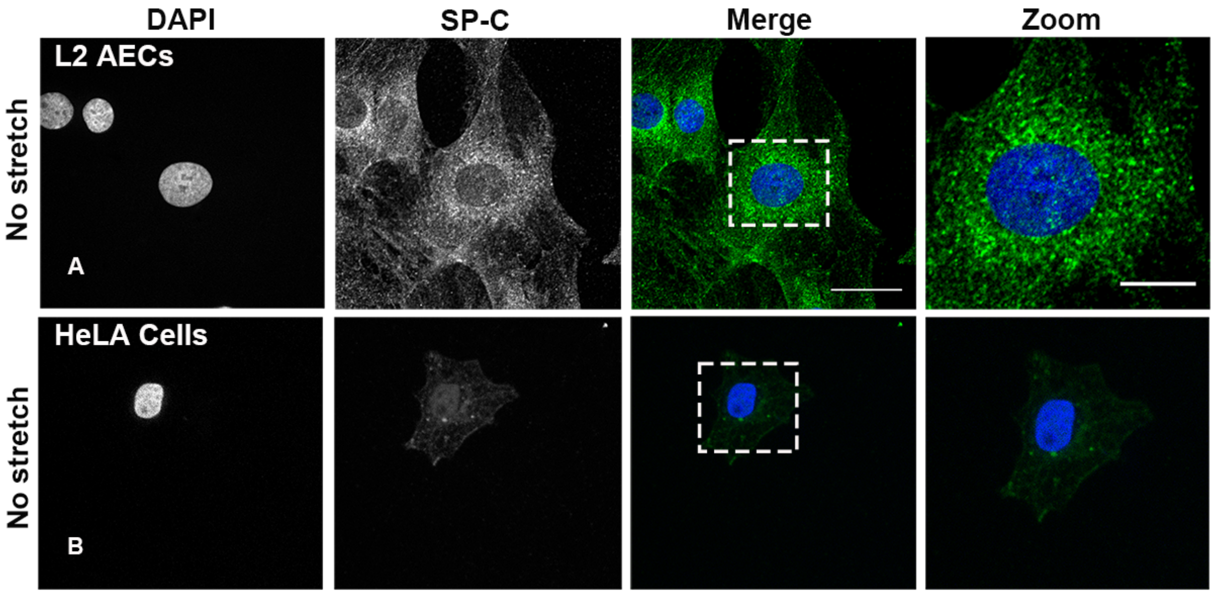

Supplementary Figure 2. Typification of the L2 AECs. (A) Non-stretched, L2 AECs, and (B) HeLa cells, cervical cancer cells, were fixed, stained with an antibody against the alveolar type II cell marker, surfactant C (SP-C), and DAPI (DNA). Confocal fluorescence microscope images were showed a single channel in grey for DAPI and SP-C; Merge: DAPI (blue), SP-C (green) as well as, magnification panel. In the merge image panel shows, a higher magnification of the region delimited by the white box. Scale bar: 10µm.
