# Supplementary material for: Static Stretch Increases the Pro-Inflammatory Response of Rat Type 2 Alveolar Epithelial Cells to Dynamic Stretch
Source: Front Physiol. 2022 Apr 11;13:838834. doi: 10.3389/fphys.2022.838834 (PMC9035495; doi:10.3389/fphys.2022.838834)
Supplement: Supplementary file 12 [file Image7.pdf]

Supplementary Material

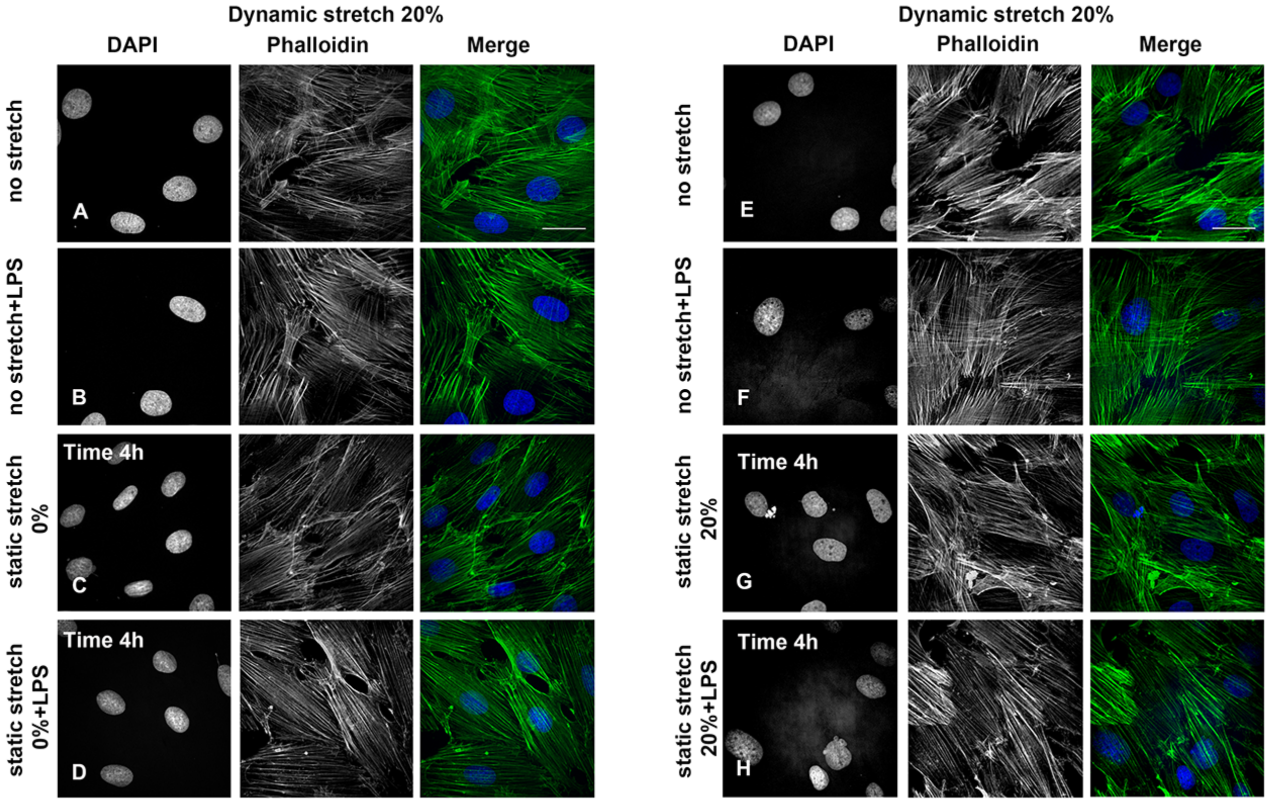

Supplementary Figure 7. Effect of static stretch combined with a dynamic stretch of 20% on L2 AECs with and without LPS. Cells were treated, fixed, stretched during 4h, stained with antibody phalloidin (actin filaments) and DAPI (DNA), then analysed by confocal fluorescence microscopy. Data are displayed as a projection of Z-sections. Images of the first and second columns are single channels in grey scale for DAPI and phalloidin; third column: Merge: DAPI (blue) and phalloidin (green) respectively. Data are displayed as a projection of 1µm Z-sections. Scale bars: 30µm.
