# Supplementary material for: Static Stretch Increases the Pro-Inflammatory Response of Rat Type 2 Alveolar Epithelial Cells to Dynamic Stretch
Source: Front Physiol. 2022 Apr 11;13:838834. doi: 10.3389/fphys.2022.838834 (PMC9035495; doi:10.3389/fphys.2022.838834)
Supplement: Supplementary file 13 [file Image1.pdf]

Supplementary Material

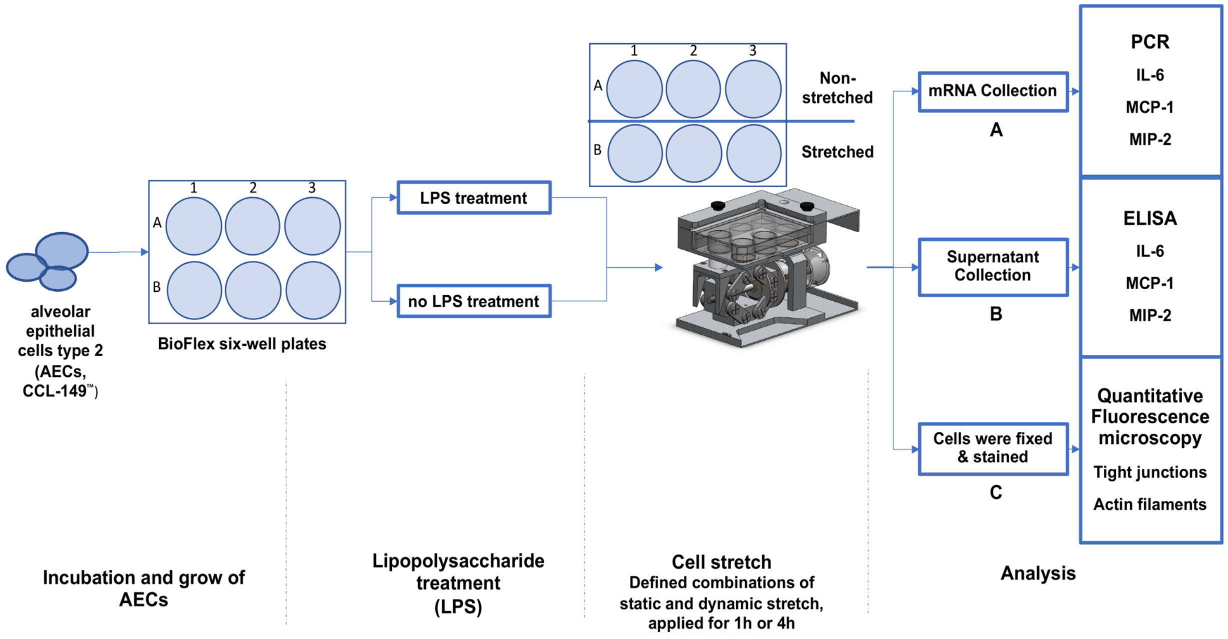

Supplementary Figure 1. Experimental design of this study. Alveolar epithelial L2 cells (AECs) were grown on BioFlex six-well plates. Cells were treated or not (untreated group) with 2 µg/ml of lipopolysaccharide (LPS) for 1h. Then, cells were stretched using a custom designed device (shown in Figure 1A), for either 1h or 4h, under different combinations of static and dynamic stretch. (A) Total cellular mRNA was collected and used to evaluate gene expression of interleukin-6 (IL-6), monocyte chemoattractant protein-1 **(**MCP-1), and macrophage inflammatory protein 2 (MIP-2) by polymerase chain reaction (PCR). (B) Cellular growth medium (supernatant) was collected and used to investigate the secretion of IL-6, MCP-1, and MIP-2 by enzyme-linked immunosorbent assay (ELISA). (C) Cells were fixed and stained with a specific antibody against Zonula Occludens Protein 2 (ZO-2) to identify tight junctions, or with phalloidin to label actin filaments. Cells were later imaged using confocal fluorescence microscopy, and images were quantitatively analyzed using ImageJ and the FiloQuant and OrientationJ plugins.
